# Education, socioeconomic status, modifiable dementia risk and cognitive performance in older adults at risk of cognitive decline: a cross‐sectional study of the FINGER‐NL trial

**DOI:** 10.1002/alz70860_101509

**Published:** 2025-12-23

**Authors:** Lion M. Soons, Kay Deckers, Marissa D. Zwan, Sophie C.P.M. Wimmers, Lisa Waterink, Nynke Smidt, Jurgen A.H.R. Claassen, Yannick Vermeiren, Wiesje M. van der Flier, Sebastian Köhler

**Affiliations:** ^1^ Alzheimer Center Limburg, School for Mental Health and Neuroscience, Maastricht University, Maastricht, Netherlands; ^2^ Alzheimer Center Limburg, Mental Health and Neuroscience Research Institute, Maastricht University, Maastricht, Netherlands; ^3^ Alzheimer Center Amsterdam, Neurology, Vrije Universiteit Amsterdam, Amsterdam UMC location VUmc, Amsterdam, Netherlands; ^4^ Department of Psychiatry and Neuropsychology, Alzheimer Centrum Limburg, Mental Health and Neuroscience Research Institute (MHeNs), Maastricht University, Maastricht, Netherlands; ^5^ Department of Epidemiology, University of Groningen, University Medical Center Groningen, Groningen, Netherlands; ^6^ Department of Geriatrics & Radboudumc Alzheimer Center, Radboud University Medical Center, Nijmegen, Netherlands, Netherlands; ^7^ Division of Human Nutrition and Health, Wageningen University & Research, Wageningen, Netherlands; ^8^ Alzheimer Center, Department of Neurology, Amsterdam UMC, Vrije Universiteit Amsterdam, Amsterdam Neuroscience, Amsterdam, Netherlands

## Abstract

**Background:**

Socioeconomic inequalities in cognitive functioning and dementia risk have been reported, which might partly act through modifiable risk factors. We investigated the association between education, socioeconomic status (SES) and modifiable dementia risk with cognitive performance in older adults at risk of cognitive decline.

**Methods:**

We used cross‐sectional data from the Dutch, randomized, controlled, multidomain lifestyle intervention FINGER‐NL multicenter trial of 1210 adults aged 60 to 79. SES was measured by equivalent household income. Modifiable dementia risk was captured with the LIfestyle for BRAin health (LIBRA) index of 12 modifiable health conditions and lifestyle factors. A total score was created from individual test results of neuropsychological test battery (NTB). Associations of SES tertiles and LIBRA with the NTB total score were investigated with multiple linear regression analyses adjusted for demographics and study site. Structural equation modelling was used to examine potential mediation by LIBRA and the role of education.

**Results:**

A total of 1048 participants (mean age 67.5, 37.9% men) with full data were included. Being in the low SES tertile was associated with poorer NTB scores, independent of demographics and LIBRA (low vs middle: B=‐0.13, 95%CI=‐0.27 to 0.01; low vs high: B=‐0.20, 95%CI=‐0.34 to ‐0.06). Individuals with low SES also had higher (=worse) LIBRA scores (low vs middle: B=0.72, 95%CI=0.33 to 1.10; low vs high: B=0.53, 95%CI=0.15 to 0.92). Similarly, low educational levels were significantly associated with worse NTB scores (low vs high B=‐0.45, 95%CI=‐0.62 to ‐0.27) and higher LIBRA scores (low vs middle: B=0.65, 95%CI=0.13 to 1.17; low vs high: B=2.01, 95%CI=1.54 to 2.48). However, LIBRA scores did not explain NTB score differences by SES or education.

**Conclusion:**

Higher SES and educational level appear protective factors for optimal cognitive performance and a more favorable risk profile, but the latter did not explain cognitive differences. Individual and community‐based prevention programs to reduce social inequalities in brain health should therefore focus on individual as well as more upstream environmental risk factors.

